# The Threat of Vector-Borne Diseases in Sierra Leone

**DOI:** 10.4269/ajtmh.22-0495

**Published:** 2023-06-05

**Authors:** Robert T. Jones, Scott J. Tytheridge, Samuel J. Smith, Rebecca S. Levine, Mary H. Hodges, Rashid Ansumana, Sophie Wulff, Jimmy Whitworth, James G. Logan

**Affiliations:** ^1^ Department of Disease Control, Faculty of Infectious and Tropical Diseases, London School of Hygiene & Tropical Medicine, London, United Kingdom;; ^2^ Arctech Innovation, The Cube, Dagenham, United Kingdom;; ^3^ Directorate of Disease Prevention and Control, Ministry of Health and Sanitation, Freetown, Sierra Leone;; ^4^ Centers for Disease Control and Prevention, Atlanta, Georgia;; ^5^ Helen Keller International Sierra Leone, Freetown, Sierra Leone;; ^6^ Mercy Hospital Research Laboratory/School of Community Health Sciences, Njala University, Bo, Sierra Leone;; ^7^ Department of Infectious Disease Epidemiology, Faculty of Epidemiology and Population Health, London School of Hygiene & Tropical Medicine, London, United Kingdom

## Abstract

Sierra Leone is vulnerable to a wide range of vector-borne diseases transmitted by mosquitoes, tsetse flies, black flies, and other vectors. Malaria, lymphatic filariasis, and onchocerciasis have posed the greatest threat and have received the most attention in terms of vector control and capacity for diagnosis. However, malaria infection rates remain high, and there is evidence of circulation of other vector-borne diseases, such as chikungunya and dengue, which may go undiagnosed and unreported. The limited understanding of the prevalence and transmission of these diseases restricts the capacity for predicting outbreaks, and impedes the planning of appropriate responses. We review the available literature and gather expert opinions from those working in the country to report on the status of vector-borne disease transmission and control in Sierra Leone, and present an assessment of the threats of these diseases. Our discussions highlight an absence of entomological testing for disease agents and the need for more investment in surveillance and capacity strengthening.

## INTRODUCTION

Sierra Leone has a tropical monsoon climate suitable for many of the vectors that transmit arboviruses, plasmodia, and parasitic worms.^1^^–^^4^ It is also a country with pervasive poverty, ranking 182nd out of 189 countries in the Human Development Index in 2020.^5^ It has a high level of illiteracy and limited access to safe drinking water, adequate sanitation, and consistently reliable power sources. It also has overcrowded housing and incomplete access to quality health services, all of which contribute to a high burden of disease.^6^^–^^8^

Malaria remains the most common cause of illness and death in the country, accounting for approximately 50% of outpatient visits and 38% of hospital admissions.^6^^,^^9^^,^^10^ The Ministry of Health and Sanitation’s (MoHS’) vector control program is estimated to have achieved reductions of less than 25% in malaria case incidence by 2020 compared with 2015 data, and 25% to 40% in malaria mortality rate. However, like much of the African region, the country appears offtrack for the WHO’s Global Technical Strategy for Malaria 2016–2030 aims for a reduction in malaria case incidence and mortality rate of at least 75% by 2025 and 90% by 2030 from the 2015 baseline.^11^^,^^12^ There are also burdens of chronic infections such as lymphatic filariasis (LF) and onchocerciasis,^13^ and threats from other vector-borne diseases (VBDs) that cause acute infections.^14^^,^^15^ A limited understanding of the prevalence and transmission of VBDs in the country restricts the capacity for predicting disease outbreaks and impedes the planning of appropriate responses. Other challenges include an inadequacy of accurate diagnoses, high mortality associated with sickle cell disease,^16^^,^^17^ and a dearth of entomological studies on the vectors themselves.

Since the Ebola epidemics in West and Central Africa in 2013 through 2016, there has been considerable investment in creating programmatic preparedness for health system responses to viral epidemics^18^ and in revitalizing the Integrated Disease Surveillance and Response System in Sierra Leone.^19^ However, such preparations do not normally include the establishment of entomological surveillance and expertise, nor the capacity strengthening needed to respond appropriately to an outbreak that is transmitted by arthropods. In this context, capacity strengthening should include provision of resources for routine surveillance, pathogen detection, and emergency control measures, not only for human disease, but also for the vectors themselves.^20^^–^^22^ As the mass drug administration (MDA) programs for onchocerciasis and LF phase out as a result of reaching elimination targets as specified in transmission assessment surveys (TASs) between 2013 and 2019, continued surveillance for these diseases will be an important element for the MoHS to adopt to avoid or control recrudescence.

Here, we present a review of the threat of VBDs in Sierra Leone, focusing first on the major vectors and diseases present in the country, followed by the efforts being undertaken to control them, and finally an appraisal of the capacity to respond.

## MATERIALS AND METHODS

### Literature search.

Literature searches were conducted using the resources of the U.S. National Library of Medicine NIH (PubMed) between November 2018 and January 2019, and again in March 2021. Searches were made sequentially for each VBD as detailed in the WHO VBD factsheet.^23^ Search terms were, therefore, disease (e.g., “chikungunya”) (All Fields) AND “Sierra Leone” (All Fields), with no restrictions on date of publication or language. Retrieved articles were screened for information on vectors, VBDs, and their control. A search result was not included in our analysis if it did not provide additional information on the distribution or threat of vectors or VBDs in Sierra Leone. For example, an article describing malaria infections in British military personnel deployed to the country would be identified through our search terms but would not contribute to our assessment of the distribution or threat of this disease. The searches were supplemented through the bibliographies and references of the articles identified, and additional online searches to include other West African countries.

### Brief entomological sampling.

Immature mosquito collection was conducted opportunistically on two occasions in January 2019 (dry season) to generate insecticide resistance data for this review. A complete submersion dipping technique was used to sample in the New Life City area, near Lumley in western Freetown (lat. 8.4614N, long. –13.2742W).^24^ Late-stage larvae and pupae were collected from stagnant pools and the edges of a slow-flowing river. The river was estimated to be no more than 0.4 m in depth and was littered with household waste. Larvae and pupae were kept in containers of their native water, covered with a net. Emerging adults were kept alive and fed a diet of 10% glucose ad libitum (Sigma-Aldrich, St. Louis, MO); their genus and sex were determined by morphology. Females age 3 to 5 days were aspirated and introduced in groups of 20 to WHO assay tubes with either control or insecticide-treated paper (Universiti Sains Malaysia) to test their resistance to permethrin, according to WHO guidelines.^25^ The mosquitoes were exposed for 1 hour, after which counts of knockdown were taken. Mortality was assessed again after 24 hours.

Adult mosquitoes were collected at a residential site on Wilkinson Road in western Freetown (lat. 8.4708N, long. −13.2744W) using a CDC light trap. The trap was placed outdoors 0.5 to 1.0 m from the ground on five nonconsecutive nights in January 2019. The trap was equipped with a lamp but no carbon dioxide source. Because of the use of window screens and air conditioning in the Public Health England property on which samples were collected, there were few mosquitoes indoors; thus, trapping was restricted to outdoors. Adults were collected between 9:00 pm and 7:30 am. The specimens from each catch were placed in a freezer, and the genus and sex of each was then determined by morphology. Adult mosquitoes were also collected in a residential area of Bo City using CDC light traps with no carbon dioxide source indoors and, on one occasion, outdoors. The entire adult mosquito collections across sampling sites included six trapping nights ranging from 10 hours 0 minute to 12 hours 45 minutes (with the earliest start time being 6:20 pm), totaling 70 hours 10 minutes of trapping time. The findings from these bioassays and mosquito collections were interpreted in the context of current vector distribution and the challenges of vector control.

### Expert opinion.

To supplement the collection of published data, we discussed the capacity of Sierra Leone to monitor and respond to the threat of VBDs with individuals associated with various organizations, including the President’s Malaria Initiative (PMI), Afro-European Medical and Research Network, Kenema Government Hospital (KGH), University of Sierra Leone, Liberty University Health Services, and George Mason University. The outcomes of these meetings are summarized herein and provide first-hand experiences of those tackling the threat of VBDs in Sierra Leone.

### Risk assessment.

**Figure 1.**
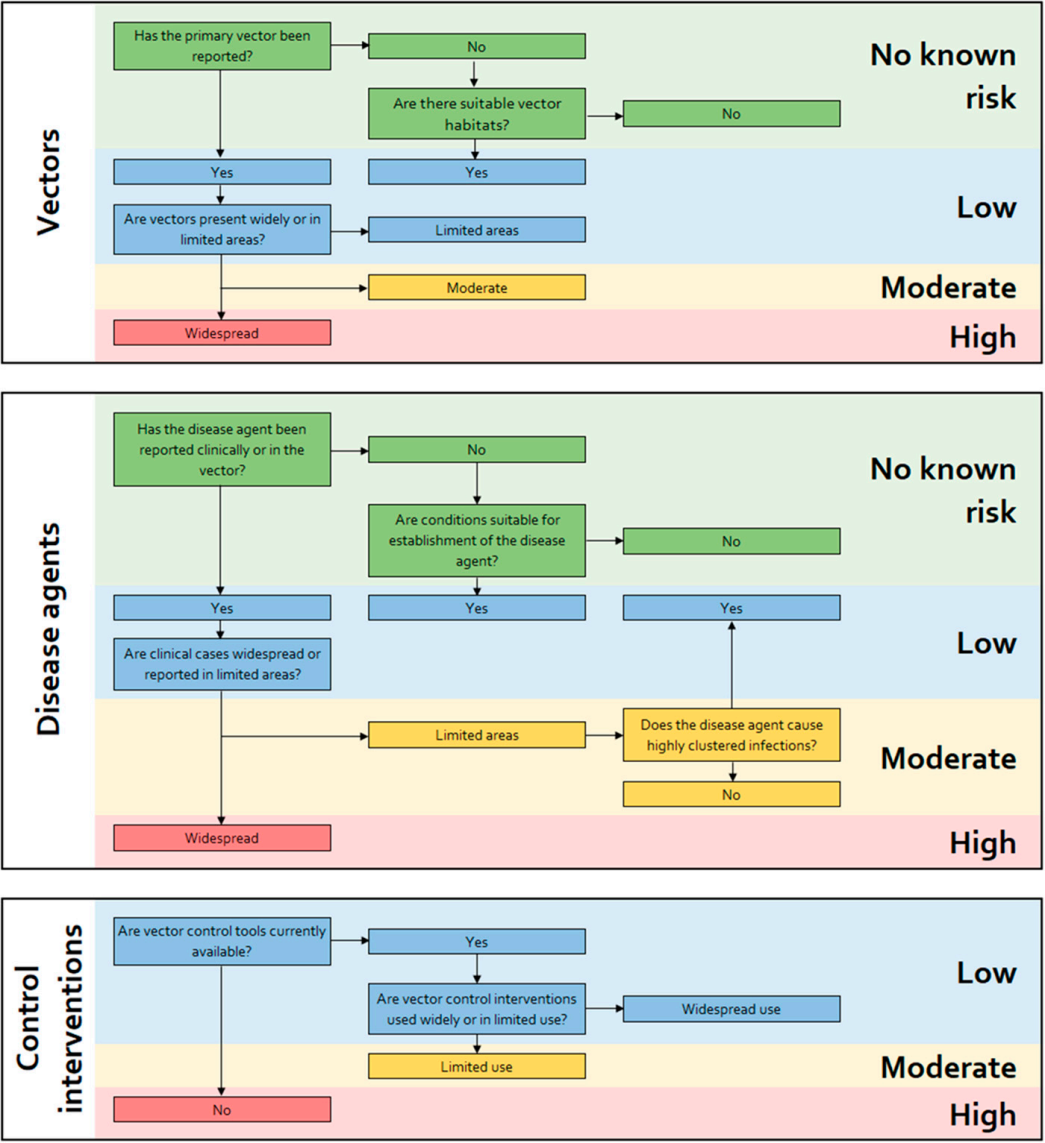
Flow chart to guide qualitative risk assessment in Sierra Leone.

The threat of VBDs in Sierra Leone was assessed through the results of the literature search and discussions. Classifications used previously to determine the global risk of selected arboviral diseases^26^ were adapted, using a framework developed specifically for the evaluation and management of health risks to the public caused by vector-borne pathogens.^27^ A flow chart showing the three categories considered in the risk assessment—vector, disease agents, and control interventions—is presented in Figure 1. The risk assessment was based on information in the articles cited within this study.

## RESULTS

### Literature search.

The literature searches returned a total of 638 articles, of which 243 (38%) were identified through the search for “Malaria” AND “Sierra Leone,” and 160 (25%) were identified through the search for “Lassa fever” AND “Sierra Leone” (Supplemental Table 1). No articles were found for searches of sand fly fever, tick-borne encephalitis, or tularemia in Sierra Leone. After removing duplicates, 537 articles remained. Information was extracted from 38 articles for the purposes of this publication.

### *Anopheles* mosquitoes.

**Figure 2.**
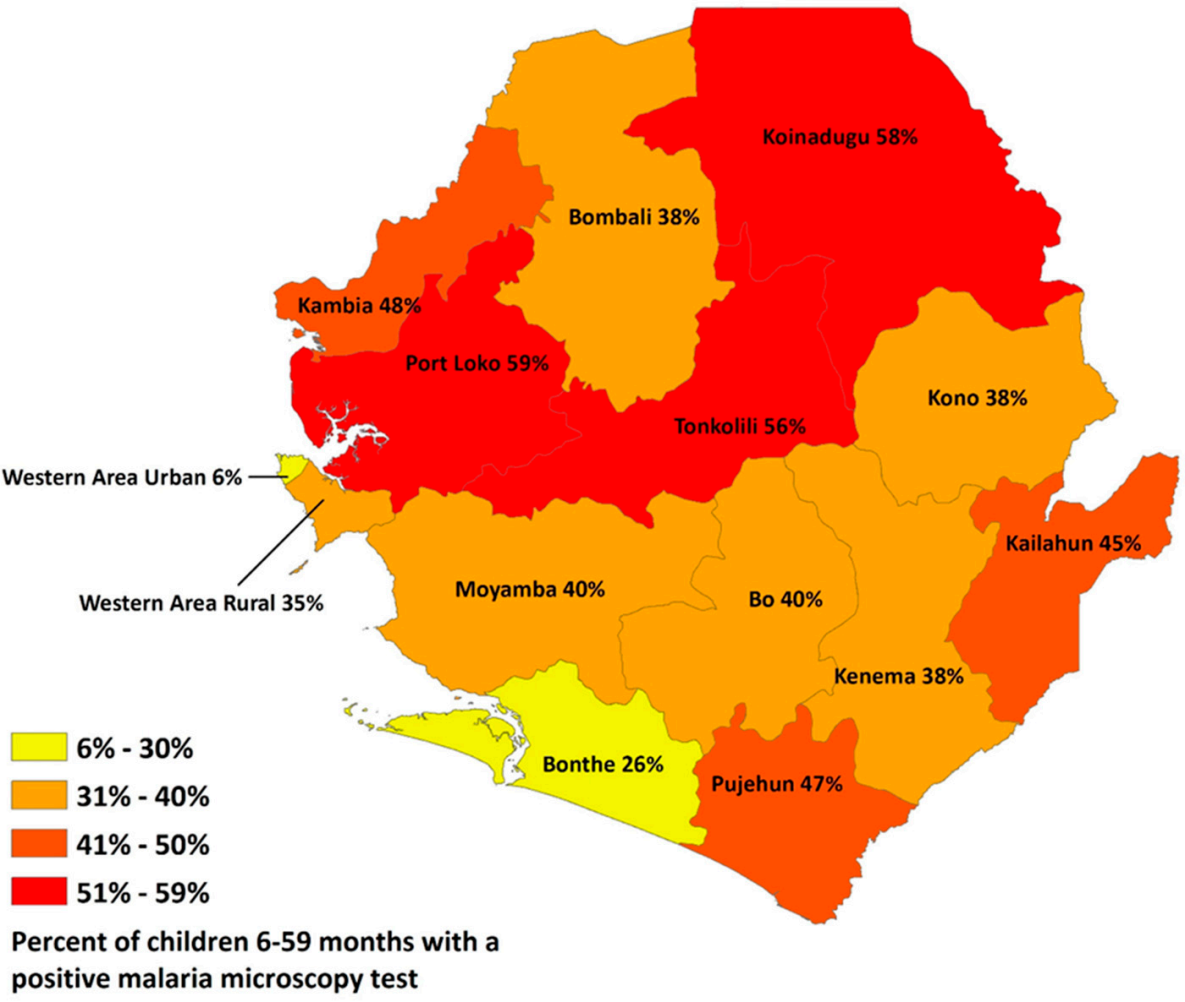
Geographic distribution of malaria parasitemia in children 6 to 59 months old, by district. Sierra Leone 2016 malaria indicator survey, sourced through the President’s Malaria Initiative, *FY 2018–2019 Sierra Leone Malaria Operational Plan*.^28^

Malaria is widespread in Sierra Leone (Figure 2^28^). Exposure begins at birth and is transmitted perennially, with little seasonal variation.^29^ There were 2,615,850 malaria cases reported nationwide in 2019, and 6,824 malaria deaths reported.^30^ Large parts of the country have more than 300 confirmed cases per 1,000 population,^31^ and the disease is a major impediment to socioeconomic development, with an estimated 7 to 12 days lost on average per episode of malaria.^11^ *Anopheles gambiae* s.s. is considered the most important vector of malaria, although studies published in 1994 revealed *Anopheles funestu*s to be a secondary dry-season vector.^3^ *Anopheles melas* has also been reported and is of lesser importance in malaria transmission.^11^

The most recent long-lasting insecticidal net (LLIN) distribution campaign in Sierra Leone, conducted by the National Malaria Control Program (NMCP) in 2020, achieved a national target of 100% population coverage (at least one net received per household).^32^ Mass distribution campaigns are expected to continue every 3 years, along with continuous distribution of free nets through antenatal clinics and expanded program of immunization visits for children younger than five years.^11^ Insecticide-treated net distribution as a means of vector control has been a key intervention promoted by the NMCP. The 2020 mass distribution campaign used LLINs containing the synergist piperonyl butoxide (PBO), consistent with data collected by the PMI showing intense pyrethroid resistance that was reduced by the addition of PBO.^33^ With this campaign, Sierra Leone became the world’s first (and only) country to provide 100% population coverage with a next-generation bed net (Dr. R. Levine, personal communication). By the end of 2020, all nets distributed through routine channels (such as expanded program of immunization visits and antenatal care) were also PBO nets, and by the beginning of 2021, the NMCP only distributed nets with this synergist. In addition, improvements in case management in health facilities and use of intermittent preventive treatment in pregnancy have been valuable malaria control tools. During and since the Ebola outbreak in 2014, intermittent preventive treatment in infancy was piloted, and Sierra Leone has become the first country to roll out intermittent preventive treatment in infancy nationwide.^34^^,^^35^ Indoor residual spraying (IRS) as a means of vector control was used in selected chiefdoms as a pilot in 2011 to 2012 using the pyrethroid lambda-cyhalothrin.^11^ An new, nonpyrethroid-based IRS program began in the Bo and Bombali districts in May 2021, using the next-generation neonicotinoid insecticide clothianidin. To inform vector control decision making, insecticide resistance monitoring of *Anopheles* mosquitoes has been conducted by the PMI since 2018, with mosquitoes originating at sentinel sites in five regionally representative districts of Sierra Leone.^33^

The parasitic worms causing LF, *Wuchereria bancrofti*, are also transmitted by mosquitoes of the *An. gambiae* complex, whereas *Culex* species are thought to have little or no role in their transmission in Sierra Leone.^36^^,^^37^ Parts of the country, historically, have had among the highest microfilariae (mf) prevalence in Africa. Mass drug administration with albendazole and ivermectin began in 2008, with the objectives of interrupting LF transmission and alleviating or preventing LF-related disability and suffering. As recommended by the WHO, TASs are conducted to determine when infections have been reduced below these target thresholds and MDA can stop. After five effective rounds of MDA, mf prevalence was reduced significantly in 8 of 12 districts co-endemic for LF and onchocerciasis (< 1% prevalence across all sites), and these districts no longer needed mass treatment. Four districts (Bombali, Koinadugu, Kailahun, and Kenema) were required to continue MDA for three additional rounds, but repeat pre-TAS monitoring found they again failed to reach the threshold of less than 2% antigenemia prevalence that would qualify them for TAS. Testing in two additional districts, that were endemic for LF but not for onchocerciasis found that the Western Area Urban qualified for conducting TAS, but the Western Area Rural failed to qualify.^38^ Reasons for failures to meet thresholds include the districts’ proximity to the borders of Guinea and Liberia, which have not yet achieved 100% MDA at baseline, greater rates of infection prior to MDA, and the migration of humans, cattle, and mosquitoes.^39^^,^^40^ The movement of individuals with LF to Bombali, Koinadugu, and the Western Area Rural for traditional management of the disease may have created foci of persistent infection.^38^ Results published in December 2020 show that 3.8 million people in Sierra Leone no longer required MDA for LF, but major challenges remain to achieve LF elimination in the entire country.^38^

### *Aedes* mosquitoes.

Entomological surveys conducted in 1972 identified *Aedes aegypti* in Sierra Leone,^41^ and more recent surveillance of *Ae. aegypti* from 2017 confirm the species continues to thrive—at least in the northern, southern, and western regions of the country^42^—if not nationwide (surveillance was not conducted in the eastern region). Our brief entomological collections identified nine *Ae. aegypti* mosquitoes in Freetown, but none in Bo City, although more extensive surveillance efforts have confirmed its presence there.^42^ Predicted distribution maps suggest that *Ae. aegypti* are particularly common in coastal regions of Sierra Leone, as well as in neighboring Guinea and Guinea-Bissau.^43^ The sister taxon *Aedes albopictus* is not believed to be present in Sierra Leone,^44^ but modeling studies predict that parts of the country are highly suitable for this species,^43^^,^^45^ and further entomological surveillance is needed.

There is evidence of infection with *Aedes*-transmitted arboviruses in Sierra Leone. In 2012 and 2013, a survey of 1,668 febrile residents in Bo revealed that 39% were positive by rapid diagnostic tests for IgM directed against chikungunya virus or a related alphavirus.^46^ This suggests a high prevalence of chikungunya infection in individuals in Bo at that time, although some of these samples may be a result of cross-reactivity with other uncharacterized alphaviruses. Furthermore, the screening of patient with suspected Lassa fever who presented at the KGH between 2011 and 2014 also revealed that a significant number had evidence of infection with chikungunya virus.^15^ More recent testing of patients diagnosed with febrile jaundice between 2016 and 2017 identified chikungunya sequence reads in serum samples from 2 of 96 patients (2.08%).^47^

Similarly, dengue virus IgG and IgM were also reported in serum samples collected from the 2011 to 2014 KGH patient cohort.^15^ These findings supported earlier studies indicating that all four serotypes of dengue virus likely circulate in Kenema and surrounding areas.^1^ Evidence of infection with an additional arbovirus—the yellow fever virus—was reported in 1975.^41^ At that time, yellow fever was found to have been in recent circulation, and most of the population had no immunity. Yellow fever vaccinations were introduced in 2003 and continue to be given to infants at 9 months of age. The most recent yellow fever outbreak was reported in 2011, when two human cases were confirmed by IgM ELISA.^48^

Zika is also an *Aedes*-borne arboviral disease. The outbreak that affected parts of South America and Asia from 2015 to 2017, and reached Cape Verde in September 2015, was not reported to have affected Sierra Leone. The WHO classifies Sierra Leone as an area with an established competent vector, but no known documented past or current transmission.^49^ Nevertheless, serological evidence from the 1970s suggested that the virus was present throughout the country at that time.^41^^,^^50^ Rift Valley fever virus can be transmitted by a range of mosquitoes in West Africa, including multiple *Aedes* and *Culex* species.^51^ Data collected from 2007 to 2014 indicate a seroprevalence for Rift Valley fever virus of 1.8% from samples submitted to the KGH from patients with symptoms resembling Lassa fever. Among these samples, there was an observed increase in prevalence from 1% to 3% in 2008 to 2012 and 11% in 2014.^14^

### *Culex* mosquitoes.

There have been no confirmed cases of Japanese encephalitis in Sierra Leone,^52^ which is not unexpected given there is no routine surveillance for this disease. The primary vector, *Culex tritaeniorhynchus*, has not been recorded in Sierra Leone, but was identified in entomological surveys in Senegal,^53^ and there are other potential vectors of Japanese encephalitis in Sierra Leone, including *Culex quinquefasciatus*.^54^ West Nile virus (WNV) has been isolated in Africa from several mosquito species, including *Culex antennatus*, *Culex univittatus*, and *Culex pipiens* in Egypt, and *Culex poicilipes* in Senegal.^55^ *Culex quinquefasciatus* is considered a potential vector to humans in West Africa, allowing for transmission in sylvatic or urban contexts, and has been widely caught in Sierra Leone as well as in neighboring countries.^54^^,^^56^ Evidence of recent exposure to WNV has been detected in serum samples collected at the KGH from patients suspected of having Lassa fever infection. Of 253 samples from patients submitted to the KGH during 2006 through 2008, seven (2.8%) were WNV IgM positive.^57^ The larger set of serum samples collected at the same hospital between 2007 and 2014 was not tested specifically for WNV; however, 330 of 624 samples (52.9%) tested positive using a pan-flavivirus assay, which was capable of detecting IgG antibodies to WNV as well as dengue, yellow fever, Japanese encephalitis, and tick-borne encephalitis virsues.^14^ There are no specific control interventions in place for *Culex* in Sierra Leone.

### Mosquito sampling.

Our brief entomological sampling found a total of 212 *Culex* (177 reared from immature stages and 35 trapped adults) in Freetown and 167 in Bo City. This compared with 26 *Anopheles* collected (as larvae/pupae) in Freetown and 38 (adults) collected in Bo City. Entomological data collected by the PMI and the CDC also indicate that *Culex* are common in the country (R. Levine, unpublished; E. Alyko, unpublished). We further found *Culex* to be resistant to permethrin [2 of 59 (3.4%) knocked down at 1 hour and 2 of 59 (3.4%) dead at 24 hours, adjusted to 0% dead using Abbott’s formula resulting from mortality of 1 of 20 (5%) in a control test], which may have been anticipated given the extensive resistance to permethrin that is seen in other mosquito species in Sierra Leone.^28^^,^^42^ We note the limitations of our small sample size and limited geographic sampling locations, and recognize that further investigations of insecticide resistance are required.

In summary, *Anopheles, Aedes*, and *Culex* mosquitoes are present in Sierra Leone. Although malaria continues to be the greatest mosquito-borne disease threat, there is recent evidence of infection with chikungunya and other arboviruses, and these pathogens likely remain both present and underreported.

### Sand flies.

In West Africa, cutaneous leishmaniasis is considered to be endemic in a belt running from Mauritania, Gambia, and Senegal in the west to Nigeria and Cameroon in the east.^58^ However, there are no confirmed reports of infections of leishmaniasis in Sierra Leone, and it is not a disease for which a control strategy is in place for the country.^59^ Sandfly fever (also called Phlebotomus fever, three-day fever, or Papatasi fever) is transmitted to humans by the bite of phlebotomine sand flies. Although sandfly fever pathogens are distributed across parts of North Africa, they are not present in West Africa.^60^^,^^61^

### Ticks.

In Sierra Leone, the tick species *Hyalomma rufipes*, *Hyalomma truncatum*, *Amblyomma variegatum*, and *Rhipicephalus evertsi* have been recorded,^62^ all four of which have been known to vector Crimean-Congo hemorrhagic fever virus (CCHFV) in other geographic regions. Ticks in the genus *Hyalomma* are particularly important vectors of CCHFV.^63^ Screening of serum samples submitted to the KGH between 2007 and 2014, primarily from Sierra Leone, but with some samples from Liberia and Guinea, detected 13 of 641 samples (2.0%) positive for CCHFV.^14^ One of 96 samples (1.0%) from febrile jaundice patients was also found to be positive for CCHFV sequence reads.^47^

*Amblyomma* ticks are also vectors of *Rickettsia.* A serosurvey for evidence of rickettsial infections conducted in rural populations of several tropical rainforest areas in the districts of Bo, Moyamba, Bonthe, and Pujehun suggested that the overall prevalence of diagnostic antibody titers to spotted fever-group rickettsiae in Sierra Leone was 5.3%.^64^ The surveyed populations were unvaccinated and included children 3 to 15 years old, and adults. By contrast, there is no evidence for the presence of *Borrelia burgdorferi*, the causative agent of Lyme disease, in sub-Saharan Africa,^65^ and the *Ixodes* vector appears to be absent in Sierra Leone.^66^ This absence also means that Sierra Leone is not at risk of tick-borne encephalitis.

The epidemiology of tularemia in Africa, a zoonotic disease caused by *Francisella tularensis*, remains unknown.^67^ Although in the northern hemisphere *F. tularensis* is transmitted by ticks,^68^ tularemia may be acquired in Africa through contaminated water and may be maintained in the environment by various terrestrial and aquatic mammals such as rabbits, hares, and water rats.^68^^,^^69^

### Tsetse flies.

Sierra Leone is endemic for *Trypanosoma brucei gambiense*, which is transmitted by *Glossina* tsetse flies and is the causative agent of human African trypanosomiasis (HAT). Species of tsetse fly known to occur in Sierra Leone include the riverine flies *Glossina palpalis palpalis*, which live in degraded forest habitats, and *G. p. gambiensis*, which live in riparian vegetation along the rivers of humid savannahs and disperse along watercourses.^70^^–^^72^ A review of progress in the elimination of HAT in 2014 determined that 117,000 people, or 2.0% of the human population, was at low or very low risk, in an area that covered 1.5% of Sierra Leone’s land area.^73^ This low-risk area is located along the northwest border with Guinea, with the mangrove habitats there having the greatest prevalence for HAT in West Africa.^74^ Although Guinea has reported an average of 74 new cases per year in the past decade, no cases of HAT have been reported in Sierra Leone in the same period.^75^^,^^76^ There is a lack of data on the transmission dynamics for HAT in Sierra Leone, but early surveys of tsetse flies found ecological restrictions of the species. For example, *G. p. palpalis* was observed not readily attacking humans, and was less numerous in settled and densely populated areas than in similar lacustrine and riverine areas farther in the bush.^72^ Such restrictions may contribute to the absence of HAT, whereas vector control activities and surveillance in Guinea are helping progress toward elimination.^76^

### Fleas.

Plague has largely disappeared from North and West Africa,^77^ and testing for the disease is limited. However, all African countries should be concerned by its possible emergence or reemergence. Neighboring Guinea reported cases of plague in 1969 and 1970, and there is evidence from Algeria and Madagascar that the disease can reemerge in areas that have long remained free of outbreaks.^78^ The abundance of rats and the easy access of fleas to human dwellings, in addition to other sociological factors, would enable transmission in Sierra Leone.^79^

### Black flies and midges.

Sierra Leone was formerly hyperendemic for *Onchocerca volvulus*, which causes river blindness and is transmitted by *Simulium damnosum* s.l.^80^ The National Onchocerciasis Control Program established in 1989 was expanded in 2007 into the national integrated Neglected Tropical Disease Program to include LF, schistosomiasis, and soil-transmitted helminths.^20^^,^^81^ After five annual rounds of MDA with ivermectin between 2005 and 2007, a significant reduction of onchocerciasis mf prevalence (from 2.6–0.3%) and mean density (from 50.9–17.59 mf/mL) was recorded in all 12 endemic districts. The threat of this disease is diminished and Sierra Leone is on course to reach elimination by 2025.^20^

*Mansonella* filarial nematodes (roundworms) are also transmitted by black flies and *Culicoides* biting midges.^82^ *Mansonella perstans* and *Mansonella streptocerca* have geographic distributions that cover Central and West Africa, and it is estimated that 600 million people live at high risk of contracting an infection.^83^^,^^84^ A cross-sectional epidemiological and clinical study of human filariasis reported in 1996 revealed the presence of *Mansonella* infections in Sierra Leone.^13^ In 2017, *M. perstans* was again identified in northern Sierra Leone during a repeat pre-TAS for LF (Y. Bah et al., National Neglected Tropical Disease Programme, unpublished).

### Aquatic snails.

Intestinal and urogenital forms of schistosomiasis, caused by trematode worms released by freshwater snails, are known to be endemic in seven of Sierra Leone’s districts.^39^^,^^85^^,^^86^ *Schistosoma haematobium* is much less prevalent than *Schistosoma mansoni*. The reasons for this are unclear and may be a result of changes in ecological habitats for the snail hosts. Annual MDA with praziquantel began in six districts in 2009 and was expanded to a seventh in 2010, targeting school-age children and at-risk adults.^87^ A sentinel site survey in 2012 showed a significant reduction [from 49.7% (95% CI, 46.2–53.3) in 2009 to 16.3% (95% CI, 14.4–18.4)] in the overall *S. mansoni* infection prevalence across 26 sites in the seven districts. The current control strategy is expected to expand the treatment coverage to include school-age children in low-endemicity districts.^87^

### Rodents.

Lassa fever cases have been reported from 10 of Sierra Leone’s 16 districts, with the southeastern part of the country traditionally considered the endemic zone.^88^ The Natal multimammate mouse, *Mastomys natalensis*, is the reservoir of Lassa virus and constituted 50% to 60% of the rodents captured in houses in Sierra Leone during a study conducted in the 1980s.^89^ Interactions with rodents are a long-standing feature of life in Sierra Leone and other parts of West Africa, and exposure inside the house, as well as through hunting, preparation, and consumption of rodents, enables transmission.^90^^,^^91^ Prevention of Lassa fever relies on promoting good community hygiene to discourage rodents from entering homes. The MoHS has partnered with the WHO, the Office of U.S. Foreign Disaster Assistance, the United Nations, and other organizations to establish the Mano River Union Lassa Fever Network to support the development of national prevention strategies and the enhancement of laboratory diagnostics.^92^

### Capacity for disease response within the health system.

Within the context of VBDs, we discussed vector control activities with stakeholders in Sierra Leone. Their comments on capacity are summarized in Supplemental Table 2 and reveal there are procedures for diagnosing and reporting VBDs, but the main focus is on malaria, LF, and onchocerciasis. These three focal areas each have large, nationwide programs that have proved successful even in the context of a relatively weak health system.

The health system of Sierra Leone has a network of public and private health facilities, and is organized into three tiers of care: 1) peripheral health units, with the extended community health worker (CHW) program; 2) district hospitals; and 3) referral hospitals. There are 40 hospitals in the country, and 1,281 peripheral health units: 577 maternal and child health posts, 343 community health posts, and 265 community health centers.^11^ Testing patients for malaria can be conducted in clinical settings, and CHWs or community heath volunteers (CHVs) also provide free malaria testing. These trained individuals can administer home-based rapid diagnostic tests, and these individuals have increased access to diagnosis for thousands of households. Interventions have been launched that allow CHWs or CHVs to provide noninjectable treatment of those who test positive for uncomplicated cases of malaria,^93^^,^^94^ whereas those with complicated malaria cases, or who test negative, are referred to a nearby hospital for advanced care.^9^^,^^95^

The ability to detect less common infections, such as those caused by arboviruses, has improved through the availability of immunoassay tests and polymerase chain reaction facilities at Mercy Hospital Research Laboratory.^2^^,^^46^ There are also diagnostic facilities at the One Health laboratory at Njala University, the CDC-supported laboratory at Njala, China CDC, and the KGH laboratory. However, funding and geographic limitations prevent these facilities from being used widely and routinely. Concerning entomological testing for VBD agents, the PMI regularly tests *Anopheles* collected in Sierra Leone for malaria parasites in laboratories outside of the country, and the CDC performs testing of limited *Aedes* mosquito samples for arboviruses in its U.S. laboratory. Aside from these activities, there is an absence of entomological testing for VBD agents, and none are performed within Sierra Leone, which limits the capacity to identify and respond to emerging threats considerably. Building this capacity at the national level is a priority for the MoH and partners.

### Threat assessment.

Risk assessments of VBDs have been adopted by public health organizations such as the WHO and the European CDC, but there is no standardized approach to ensuring that sufficient information is gathered for such assessments.^96^ Schmidt et al.^27^ presented a systematic approach for the analysis, assessment, and governance of emerging health risks attributed to VBDs by adapting a framework developed by the International Risk Governance Council. Their approach centers on five elements—specifically, preassessment, appraisal, characterization and evaluation, management directives, and communication—recognizing the complexity of all interacting risk factors that affect the development of these diseases. Factors that affect the probability of humans being infected by vector-borne pathogens include those associated with the vector (such as vector competence, infection rate, behavior, and distribution), the pathogen (genetic diversity, pathogenesis, transmission efficiency), and the human (behavior, control measures, severity of disease, susceptibility, and immune response). Predicting the risk of VBD cases or outbreaks is, therefore, extremely challenging, and is complicated in low-resource settings by lack of sufficient data or uncertainty of information. Furthermore, the possibility of risk altering over time as a result of climate and environmental changes, fluctuating human living habits, agricultural land use, individual human behavior, and the spread of vectors by human travel and global trade exacerbates the complications.^27^^,^^97^^,^^98^

The risk of VBDs in Sierra Leone has been assessed here according to the flow chart in Figure 1, and findings are shown in Table 1.^99^^–^^108^ In addition to the high risk of malaria, to which the country’s entire population of 7.8 million people is at risk,^109^ the arboviruses dengue, chikungunya, and yellow fever have emerged as moderate/high risks. There is historical evidence of these diseases from serological assays, and human demographic and behavioral factors may increase their incidence in the future.^110^ Lassa has also emerged as a disease of moderate to high risk. The ability to diagnose Lassa is well established in Sierra Leone, but progress is needed to prevent infections from occurring. Prevention is especially critical in recently deforested settings, where communities are less aware of the risks posed by rats and the recommendations for avoiding direct and indirect contact with their urine.

**Table 1:** Summary of vectors present and assessed threat of vector-borne disease risk in Sierra Leone

Vector	Disease	Vector present	Disease risk	Citations
*Aedes* mosquitoes	Chikungunya	*Aedes aegypti*	Moderate/high	Boisen et al.,^15^ Kraemer et al.^43^
	Dengue fever	*Ae. aegypti*	Moderate/high	Boisen et al.,^15^ Kraemer et al.^43^
	Yellow fever	*Ae. aegypti*	Moderate/high	Robin and Mouchet,^41^ Kraemer et al.^43^
	Rift Valley fever	*Ae. aegypti*	Moderate	Kraemer et al.,^43^ WHO^99^
	Zika	*Ae. aegypti*	Moderate	Robin and Mouchet,^41^ Kraemer et al.^43^
*Anopheles* mosquitoes	Malaria	*Anopheles gambiae, Anopheles funestus, Anopheles melas*	High	WHO,^100^ National Malaria Control Programme^101^
	Lymphatic filariasis	*A. gambiae*	Low	de Souza et al.^54^
*Culex* mosquitoes	West Nile virus	*Culex quinquefasciatus*	Moderate	Boisen et al.,^15^ Samy et al.^56^
	Japanese encephalitis	–	No known risk	Mackenzie et al.^52^
Sand flies	Leishmaniasis	*Phlebotomus* spp.	Low	Boakye et al.^58^
	Sandfly fever (Phlebotomus fever)	–	No known risk	Tufan and Guven^60^ Alkan et al.^61^
Ticks	Crimean–Congo hemorrhagic fever	*Hyaloma* spp.	Moderate	Boisen et al.^15^
	Rickettsial diseases	*Amblyomma variegatum*	Low	Redus et al.^64^
	Tularemia	–	Low	Petersen and Schriefer^102^
	Lyme disease and tick-borne encephalitis	–	No known risk	Ministry of Health and Sanitation, Government of Sierra Leone^59^
	Tick-borne encephalitis	–	No known risk	Lindquist and Vapalahti^66^
Tsetse flies	Sleeping sickness (African trypanosomiasis)	*Glossina morsitans*	Low	Cecchi et al.,^103^ Dunn and Adigun^104^
Fleas	Plague	–	Low	WHO^105^
	Rickettsiosis	–	Low	Dupont et al.^106^
Black flies and midges	Onchocerciasis (river blindness)	*Simulium* spp.	Low	Koroma et al.^20^
	Mansonellosis	*Culicoides* spp.	Low	Ta-Tang et al.^83^
Aquatic snails	Schistosomiasis (bilharziasis)	*Bulinus globosus, Biomphalaria pfeifferi*	Moderate	Hodges et al.,^39^ Doumenge et al.^107^
Lice	Typhus and louse-borne relapsing fever	*Pediculus* spp.	Low	Gratz^108^
Rodents	Lassa fever	*Mastomys natalensis*	Moderate/high	O’Hearn et al.^14^

WHO = World Health Organization.

## DISCUSSION

Malaria remains the primary VBD risk and is the most common cause of illness and death in Sierra Leone.^6^ Therefore, *Anopheles* mosquitoes have received the most attention in terms of both entomological monitoring and vector control. As with other sub-Saharan African countries, LLINs have been the primary tools used by the NMCP, although IRS was reintroduced in two districts in 2021. Larval source management and house improvements are both options that the MoHS and stakeholders are invited to explore in the future, but may be unrealistic in the near term and will require further evaluations within the Sierra Leonean context.^111^ Lymphatic filariasis, also transmitted by *Anopheles* mosquitoes, has an extensive elimination program centered around annual MDA that is controlling this disease effectively.

Other VBDs that have been reported in the country include those transmitted by *Aedes* and *Culex* (chikungunya, dengue, yellow fever, and Rift Valley fever viruses), ticks and fleas (spotted fever-group rickettsiae), black flies and midges (onchocerciasis), aquatic snails (schistosomiasis), and rodents (Lassa fever). The surveillance and control of these vectors are far from reaching their full potential, and there is a lack of investment in interventions suitable for *Aedes* or *Culex*, which are known to be present and are implicated in disease transmission. Options for the control of *Aedes* mosquitoes include larval control, such as environmental management and source reduction, which has also been used to control *Culex* in some settings,^112^^,^^113^ and window screens. However, there are no central programs for the control of these mosquitoes,^112^ and there are few studies on the efficacy of these types of interventions in African settings beyond historical reports.^114^ Should there be a need to control sand flies, residual insecticides may be a suitable intervention.^115^ In addition, there might be possibilities for exploiting those activities already set up for malaria control, and indeed these activities may already be having an effect on sandfly populations, as has been reported in Mali.^116^ When outbreaks of leishmaniases have occurred elsewhere in West Africa, control efforts have remained limited to the provision of care to those infected.^58^^,^^117^^,^^118^ Further investment in both entomological and epidemiological surveillance is required to understand the current, actual VBD burden and how it can be addressed. Such activities will better reveal the capacity strengthening needed to contend with VBDs and to help avoid unsuccessful vector control attempts.

The WHO Global Vector Control Response advocates for the realignment of national programs to optimize implementation of interventions against *multiple* vectors and diseases to maximize the impact of available resources.^119^ Many existing vector control interventions are known to be effective against multiple diseases, so combining vector control programs to tackle several diseases simultaneously could offer more cost-effective and sustainable disease reductions.^120^ In Sierra Leone, malaria control is currently very focused on specific interventions that target nighttime biting mosquitoes inside of homes, which are of limited value against several of the other vectors described here. The IRS implementation in 2021 may reach more vectors, but ultimately its impact may be temporary if resistance to the active ingredient develops. More holistic approaches, which include the modification of houses with window screens, ceilings, closed eaves and self-closing ventilated doors, coupled with improvements to sanitation and piped water supplies, are expected to have wide-reaching and long-lasting effects.^121^^–^^124^ To achieve such integrated vector management, there remains a need to improve knowledge of the distributions of disease and major vectors, to characterize the impacts of specific interventions, and to develop detailed plans and capacity for integrated VBD surveillance, prevention, and outbreak response.^125^

Our study has limitations, including those mentioned earlier relating to the small sample size and finite geographic coverage of our mosquito sampling, and with regard to access to data. For example, the lack of collections of *Aedes* mosquitoes or screening for arboviruses make it difficult to assess the threat of these pathogens to Sierra Leone. Furthermore, we did not receive responses from some organizations working in Sierra Leone that we contacted, such as the Chinese CDC, so our interpretations have been made in the absence of any additional information they may have collected or experiences they have gathered in the country.

## CONCLUSION

This study suggests that malaria remains the major VBD threat in Sierra Leone, and continues to receive the most attention. Our review of the literature and key informant interviews highlighted potential threats of other diseases, and an absence of entomological surveillance and testing for disease agents. Greater investment in surveillance is needed to characterize these threats more completely, and should be coupled with appropriate control interventions and investment in capacity strengthening.

## Supplemental Material


Supplemental materials
