# Supplementary material for: The Threat of Vector-Borne Diseases in Sierra Leone
Source: Am J Trop Med Hyg. 2023 Jun 5;109(1):10–21. doi: 10.4269/ajtmh.22-0495 (PMC10323989; doi:10.4269/ajtmh.22-0495)
Supplement: Supplementary file 1 [file tpmd220495.SD1.pdf]

Supplementary Table 1.

Search term	Number of articles
Chagas disease	1
Chikungunya	8
Crimean-Congo haemorrhagic	6
fever Dengue fever	13
Japanese encephalitis	2
Lassa fever	160
Leishmaniasis	1
Lyme disease	1
Lymphatic filariasis	18
Malaria	243
Mansonellosis	1
Onchocerciasis	100
Plague	3
Rickettsial diseases	6
Rickettsiosis	2
Rift Valley fever	5
Sand fly fever	0
Schistosomiasis	34
Human African trypanosomiasis	6
Tick-borne encephalitis Tularaemia	0
Typhus	0
West Nile virus	2
Yellow fever	5
Zika	16
	5
**Total**	**638**

Supplementary Table 2

Name	Affiliation	Comments on capacity
Dr. S. Smith	MoHS	Dr. Smith, the manager for the National Malaria Control Program, explained that the MoHS has many partners, including the Ministry of Environmental Health and Sanitation, PMI, CDC, Chinese Center for Disease Control and Prevention (China CDC), and Heath Security Health Directorate, and there is dialogue between the MoHS and all of these groups. For VBDs, a technical working group for Integrated Vector Management exists, but Dr. Smith conceded that it struggles to function due to the number of partners working together.<br><br>The Ebola outbreak in 2014-2015 identified a lack of surveillance activities, including for VBDs, and since then several improvements have been implemented including the preparation of weekly epidemiological reports. The MoHS is now focusing on improving data quality, as well as improving the number and specialisms of personnel. They currently have two entomologists, but benefit from entomology expertise within PMI-VectorLink. A sustainable, high-quality insectary facility is now fully-functional in Makeni district, where a diagnostic laboratory and MoHS offices are also based. This has proved highly useful to the MoHS, particularly for malaria vector surveillance and as a dedicated facility for insecticide resistance testing. It is largely available through collaboration and funding from the PMI and CDC advisors. It is suitable for work on malaria and NTDs, and has space for expansion in the future. Whilst dengue diagnostic RDTs are not currently available, it is intended that these will be purchased, albeit in limited numbers.<br><br>Procedures are in place for diagnosing and reporting VBDs, but there is limited capacity and in those areas that do not have sufficient equipment for diagnosis, cases can remain undiagnosed and unreported. A further gap is the mapping of diseases and vectors in Sierra Leone; members of the MoHS, HKI and PMI-VectorLink recognise the value of having more detailed maps of which vectors are where, such that control interventions can be appropriately targeted.

Dr. M. Hodges	Helen Keller International	Dr. Mary Hodges provides technical support to the NTDP implemented by the MoHS. This programme was initiated with financial support from the United States Agency for International Development (USAID) and other partners to eliminate onchocerciasis, and lymphatic filariasis and control schistosomiasis and STHs. It uses the integrated preventive chemotherapy strategy according to WHO guidelines<br><br>HKI train teams of young graduates who support the government in implementing the programme, and independent monitors to evaluate activities. There are 44 staff at HKI, of which nine are working with the NTDP. HKI supported the Ebola outbreak in the Western Area with the quarantine process and contact tracing. HKI introduced GPS and mobile reporting for case tracking which could be rapidly scaled-up and adapted again to meet new challenges of another VBD outbreak.
Dr. R. Levine, Mr. C. Sandi	United States Centers for Disease Control and Prevention	Dr. Rebecca Levine (Senior Research Scientist) and Mr. Christopher Sandi (Guest Researcher and Emory University Foege Fellow) on the Integrated Vector Management (now called Insecticide Resistance and Vector Control) Team at CDC, explained that following the Zika outbreak in the Americas and detection of the American Zika strain in Cabo Verde in 2015, there was concern about continuing spread of Zika to West Africa. With this worry, in conjunction with long-neglected *Aedes* entomological capacity in the region, CDC supported *Aedes* entomological training and sampling. In Sierra Leone in 2017, CDC conducted entomological training with the MoHS, led a 6 week project involving *Aedes* collection and screening for arboviruses, and hosted a multinational workshop on *Aedes* surveillance for 13 West African nations.<br><br>The six-week *Aedes* project was led by Mr. Sandi from June-August 2017, and examined sites in Bo and Bombali districts for prevalence, abundance and density of *Aedes*. Larval habitats were identified to calculate larval indices and eggs and adults were collected and sent to the US for insecticide resistance and arbovirus testing, The results were reported to the MoHS, and will allow them to make decisions on which types of insecticides could be used effectively in the future should there be a need, and which types of habitats to target for larval source reduction. The input of CDC has therefore

		added an element of *Aedes* research to that conducted on *Anopheles* for the NMCP. Dr. Levine said that while entomology in Sierra Leone has become more robust under the CDC and PMI activities, resource-constraints made it a previously overlooked field and little laboratory capacity currently exists in-country for pathogen-testing from entomological samples.
Dr. E. Alyko<br>Dr. D. Schnabel	President's Malaria Initiative (PMI)/VectorLink Project	Sierra Leone became a PMI focus country in 2017. The initiative supports key malaria interventions that are aligned with the NMCP national strategic plan to achieve access to malaria control interventions for all and reduce malaria morbidity and mortality. For prompt diagnosis and effective treatment using the "test-treat-and-track" practice, PMI procures diagnostic tests, anti-malarials, and injectable artesunate for severe disease. PMI also supports health worker training and mentoring, entomological surveillance, social and behavioural change strategies, and routine health information systems at the chiefdom level.[1]<br><br>PMI works through the VectorLink project in Sierra Leone to analyse the suitability of insecticides for use in bed nets, and determine which vector control strategies will be most effective given the results. They try to strengthen the country's capacity, and trained 12 people in entomology (including on mosquito collection and insecticide-resistance testing) at the NMCP in both 2018 and 2019. In addition, PMI conducts entomological monitoring activities at two sentinel sites (one rural and one rural/peri-urban) in each of four districts: the Western Area Rural district (representing the coastal areas of the country), Bo (representing the south), Bombali (representing the north), and Kono (where there is large-scale mining activity, representing the eastern part of the country) (67). Each month, mosquitoes are collected through human landing catches (HLC), CDC light traps placed indoors, and spraying with pyrethroid (pyrethrum spray catches). GPS coordinates of the collection sites are recorded. These activities began in May 2018.<br><br>PMI-VectorLink also rears field-collected F1 mosquitoes for use in insecticide resistance testing at the National Vector-Borne Disease Laboratory and Insectary (which PMI helped install) in Makeni, Bombali district. They have a laboratory technician, a field technician, and a coordinator, and all analysis is done in country. At the time of meeting, no PCR facilities exist in-country for molecular analyses that support detection of resistance mechanisms and

		species determination, however, the samples are sent to a laboratory in Cameroon for testing. The *Aedes*, *Culex* and *Mansonia* they collect are counted as 'Cuilicines', but it is beyond the remit of their malaria control work to analyse these specimens.
Dr R. Ansumana	Njala University/Mercy Hospital Research Laboratory	Dr. Rashid Ansumana is Dean of the School of Community Health Sciences at Njala University and head of a laboratory at Mercy Hospital, which focuses on the diagnosis of viruses and bacteria in patients, and they have an automated PCR Biofire device with different panels for analysis of samples from patients with sepsis, gastroenteritis and haemorrhagic fevers. Their multiplex system can identify malaria, flaviviruses, alphaviruses, and *Burkholderia* infections.<br><br>Whilst the laboratory therefore has the capability to detect a range of viruses transmitted by arthropods, it cannot specifically detect RVFv and is employed for the detection of infectious agents in human samples, rather than in vectors. Dr. Ansumana stated that, in his research projects, there is often funding for the study of a particular disease, but this does not allow for further analysis of other pathogens, and he sees a significant gap in the identification of pathogens in vectors. Further, without the weight of evidence it is difficult for his team to apply for grants that would allow them to specifically look for less well-known VBDs in human or mosquito samples. Funding for his work often comes from outside Sierra Leone, and is obtained in partnerships with other institutions such as George Mason University in Virginia.<br><br>When diagnoses are made from patient samples at Mercy Hospital, reports are sent to the District Health Management Team (DHMT). The hospital has sent two people to the CDC facility in Fort Collins, Colorado, for training in diagnostic methodology for Yersinia pestis. They also intend to purchase a MinION portable real-time device for DNA and RNA sequencing, which will enhance their ability to diagnose VBDs in patients.
	University of Sierra Leone	Prof. Sahr, Vice Chancellor and Principal of the University of Sierra Leone, is a professor of medical microbiology who collaborates with other scientists in Sierra Leone on work related to Ebola, Lassa, malaria, HIV and tuberculosis. The University of Sierra Leone contributes to capacity building through offering courses in public health in the College of Medicine and Allied Health Sciences. It has colleges in Freetown and Njala, and offers courses in pure

	and applied sciences, including community health services and social sciences. Their Masters in Public Health programme includes core modules in epidemiology, health promotion, and disease detection, prevention and control. Students also have the opportunity to take a module in communicable diseases, which includes content on vector-borne diseases, but he noted that there has been very little taught content on vector-borne diseases so far. The King’s College London King’s Sierra Leone Partnership is working with all faculties to provide support across medicine and nursing, but has little involvement with education around vector-borne diseases.<br><br>Prof. Sahr feels that entomology training is lacking in the College of Medicine and University of Sierra Leone, and is not currently seen as important as diagnostics. Prof. Sahr said that the Ebola outbreak has encouraged capacity building and the country is now better able to respond because of the possibility of Ebola returning. However, the mainstream hospitals are not adequate and require outbreak centres instead.
Kenema Government Hospital	The Lassa fever program at the Kenema Government Hospital in eastern Sierra Leone provides diagnostic services and clinical care for more than 500 suspected Lassa fever cases per year. The hospital has also been involved in surveillance of other viral infections to better define the nature and extent of viral pathogens infecting the Sierra Leonean population, the results of which suggest that unrecognized outbreaks of viral infection have occurred.<br><br>The Lassa ward has a triage structure for febrile illness or viral haemorrhagic fever with an ambulance system, and receives referrals from other hospitals. Dr. Samuels, Senior Clinician at Kenema Government Hospital, explained that they have RDTs for Lassa IgG and IgM, and local teams are being trained in their use, but they are not confirmatory; ELISA or RT PCR are used for confirmation of diagnosis. The PCR RDTs panel contains primers for other filoviruses so can provide differential diagnoses, and if the results are negative they have access to an Illumina MiSeq for sequencing. However, due to budgets not all samples are sequenced.<br><br>A surveillance team, from the Lassa ward and DHMT, partners with an outreach team to go out into communities for community mobilisation and

		education about disease prevention and to identify disease transmission routes. The Lassa hospital also has strong links with Tulane University School of Medicine. Virologists from Tulane have been researching Lassa fever in West Africa for more than 15 years, and with local partners have built research facilities.
Dr. K. Jacobsen	George Mason University	Dr. Jacobsen, a professor specializing in global health epidemiology, has held a long-running partnership with Njala University (Dr. Rashid Ansumana), Mercy Hospital, and the U.S. Naval Research Lab (NRL) testing new methods for infectious disease surveillance. She told us that the US Department of Defense (DoD) was supporting development and testing of diagnostic test kits tests for pathogens such as Ebola, yellow fever, and various adenoviruses and flaviviruses in several countries, including Sierra Leone.[2] Ongoing work by Dr. Jacobsen and her collaborators should provide further insight into current situation of VBDs in Sierra Leone, and by partnering with Mercy Hospital it is hoped that there will be both a transfer of skills and further investment in the facilities managed by Dr. Ansumana.

REFERENCES

1. President's Malaria Initiative, 2018. President's Malaria Initiative: Sierra Leone Country Profile. Available at: Available at: https://www.pmi.gov/where-we-work/sierra-leone/. Accessed August 5, 2021.
2. Mulvaney SP, et al., 2018. Rapid design and fielding of four diagnostic technologies in Sierra Leone, Thailand, Peru, and Australia: successes and challenges faced introducing these biosensors. *Sens Biosensing Res 20:* 22–33.
